# Structure, function, and control of the human musculoskeletal network

**DOI:** 10.1371/journal.pbio.2002811

**Published:** 2018-01-18

**Authors:** Andrew C. Murphy, Sarah F. Muldoon, David Baker, Adam Lastowka, Brittany Bennett, Muzhi Yang, Danielle S. Bassett

**Affiliations:** 1 Department of Bioengineering, University of Pennsylvania, Philadelphia, Pennsylvania, United States of America; 2 Perelman School of Medicine, University of Pennsylvania, Philadelphia, Pennsylvania, United States of America; 3 Department of Mathematics, University of Buffalo, Buffalo, New York, United States of America; 4 Department of Electrical and Systems Engineering, University of Pennsylvania, Philadelphia, Pennsylvania, United States of America; 5 Haverford College, Haverford, Pennsylvania, United States of America; 6 Philadelphia Academy of Fine Arts, Philadelphia, Pennsylvania, United States of America; 7 Applied Mathematical and Computational Science Graduate Group, University of Pennsylvania, Philadelphia, Pennsylvania, United States of America; 8 Department of Neurology, University of Pennsylvania, Philadelphia, Pennsylvania, United States of America; University of Oxford, United Kingdom of Great Britain and Northern Ireland

## Abstract

The human body is a complex organism, the gross mechanical properties of which are enabled by an interconnected musculoskeletal network controlled by the nervous system. The nature of musculoskeletal interconnection facilitates stability, voluntary movement, and robustness to injury. However, a fundamental understanding of this network and its control by neural systems has remained elusive. Here we address this gap in knowledge by utilizing medical databases and mathematical modeling to reveal the organizational structure, predicted function, and neural control of the musculoskeletal system. We constructed a highly simplified whole-body musculoskeletal network in which single muscles connect to multiple bones via both origin and insertion points. We demonstrated that, using this simplified model, a muscle’s role in this network could offer a theoretical prediction of the susceptibility of surrounding components to secondary injury. Finally, we illustrated that sets of muscles cluster into network communities that mimic the organization of control modules in primary motor cortex. This novel formalism for describing interactions between the muscular and skeletal systems serves as a foundation to develop and test therapeutic responses to injury, inspiring future advances in clinical treatments.

## Author summary

While network science is frequently used to characterize networks from genomics, proteomics, and connectomics, its utility in understanding biomechanics, orthopedics, and physical therapy has remained largely unexplored. Indeed, current clinical practice and knowledge regarding the musculoskeletal system largely focuses on single areas of the body, single muscles, or single injuries and therefore remains agnostic to mesoscale or global features of the body’s architecture that may have critical implications for injury and recovery. We addressed this gap by representing the musculoskeletal system as a graph or network, in which we considered bones and the muscular connections between them. By modeling muscles as springs and bones as point masses, we developed a perturbative approach to interrogate the function of this network. Employing this model, we calculated the network level effects of perturbing individual muscles. Using this formalism, we are able to draw new parallels between this system and the primary motor cortex that controls it, and illustrate clinical connections between network structure and muscular injury.

## Introduction

The interconnected nature of the human body has long been the subject of both scientific inquiry and superstitious beliefs. From the ancient humors linking heart, liver, spleen, and brain with courage, calm, and hope [1] to the modern appreciation of the gut–brain connection [2], humans tend to search for interconnections between disparate parts of the body to explain complex phenomena. Yet, a tension remains between this basic conceptualization of the human body and the reductionism implicit in modern science [3]. An understanding of the entire system is often relegated to a futuristic world, while individual experiments fine-tune our understanding of minute component parts.

The human musculoskeletal system is no exception to this dichotomy. While medical practice focuses in hand, foot, or ankle, clinicians know that injuries to a single part of the musculoskeletal system necessarily impinge on the workings of other (even remotely distant) parts [4]. An injury to an ankle can alter gait patterns, leading to chronic back pain; an injury to a shoulder can alter posture, causing radiating neck discomfort. Understanding the fundamental relationships between focal structure and potential distant interactions requires a holistic approach.

Here, we detail such an approach. Our conceptual framework is motivated by recent theoretical advances in network science [5], which is an emerging discipline built from an ordered amalgamation of mathematics (specifically, graph theory [6]) and physics (specifically, statistical mechanics [7]), computer science, statistics [8], and systems engineering. The approach simplifies complex systems by delineating their components and mapping the pattern of interactions between those components [9]. This representation appears particularly appropriate for the study of the human musculoskeletal system, which is composed of bones and the muscles that link them. In this study, we used this approach to assess the structure, function, and control of the musculoskeletal system.

The use of network science to understand the musculoskeletal system has increased in recent years [10]. However, the framework has largely been employed to investigate the properties of local muscle or bone networks. For example, the local structure of the skull has been examined to investigate how bones can be categorized [11]. Additionally, studies of the topology of the musculoskeletal spine network have been conducted to evaluate stresses and strains across bones [12]. A few studies do exist that address the entire musculoskeletal system, although they do not use the mathematical tools that we employed here [13,14]. The current study differs from previous work in its assessment of the entire musculoskeletal system combined with the mathematical tools of network science.

Within this broader context, we focused on the challenge of rehabilitation following injury to either skeletal muscle or cerebral cortex. Direct injury to a muscle or associated tendon or ligament affects other muscles via compensatory mechanisms of the body [15]. Similarly, loss of use of a particular muscle or muscle group from direct cortical insult can result in compensatory use of alternate muscles [16,17]. How the interconnections of the musculoskeletal system are structured and how they function directly constrains how injury to a certain muscle will affect the musculoskeletal system as a whole. Understanding these interconnections could provide much needed insight into which muscles are most at risk for secondary injury due to compensatory changes resulting from focal injury, thereby informing more comprehensive approaches to rehabilitation. Additionally, an understanding of how the cortex maps onto not only single muscles but also groups of topologically close muscles could inform future empirical studies of the relationships between focal injuries (including stroke) to motor cortex and risk for secondary injury.

## Materials and methods

### Network construction

**Fig 1:**
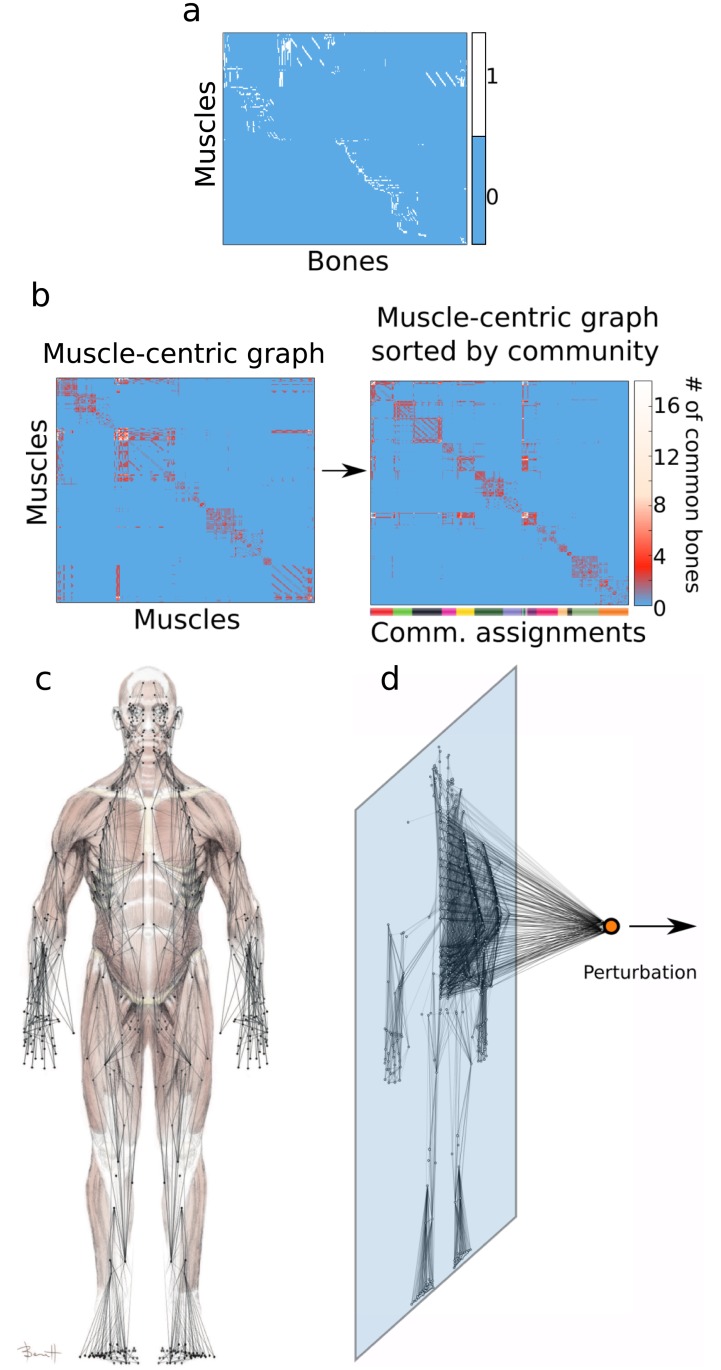
Schematic of data representations and computational methods. (a) The musculoskeletal network was first converted to a bipartite matrix, where 1/0 indicates a present/absent muscle±bone connection. (b) Communities of topologically related muscles are identified by (1) transforming the hypergraph to a muscle±muscle graph, in which each entry encodes the number of common bones of each muscle pair, and (2) subsequently, muscles were broken into communities, in which constituent members connected more densely to other members within their community than to members in other communities. (c) To facilitate perturbations, the musculoskeletal network was physically embedded, such that bones (nodes) are initially placed at their correct anatomical positions. (d) To understand the impact of single muscles on the interconnected system, all nodes linked by a selected hyperedge were perturbed in a fourth spatial dimension.

Using the Hosford Muscle tables [18], we constructed a musculoskeletal hypergraph by representing 173 bones (several of these are actually ligaments and tendons) as nodes and 270 muscles as hyperedges linking those nodes (muscle origin and insertion points are listed in S9 Table). This hypergraph can also be interpreted as a bipartite network, with muscles as one group and bones as the second group (Fig 1a). The 173 × 270 incidence matrix C of the musculoskeletal network is thus defined as C_ij_ = 1 if v_i_ ∈ e_j_ and 0 otherwise, where V = {v_1_, · · ·, v_173_} is the set of nodes (bones) and E = {e_1_, · · ·, e_270_} is the set of hyperedges (muscles). This hypergraph representation of the body eliminates much of the complexity from the musculoskeletal system, encoding only which muscles attach to which bones. All analysis was applied to only one half (left or right) of the body, because each cerebral hemisphere controls only the contralateral side of the body. Therefore, we further simplified our model by assuming left–right symmetry; in any figures in which both halves of the body are shown, the second half is present purely for visual intuition.

The bone-centric graph A and muscle-centric graph B (Fig 1b) are simply the one-mode projections of C. The projection onto bones is A = C^T^C, and the projection onto muscles is B = CC^T^. Then, the diagonal elements were set equal to zero, leaving us with a weighted adjacency matrix [5]. We obtained estimated anatomical locations for the center of mass of each muscle (and bone) by examining anatomy texts [19] and estimating x-, y-, and z-coordinates for mapping to a graphical representation of a human body (Fig 1c).

### Calculation of impact score

To measure the potential functional role of each muscle in the network, we used a classical perturbative approach. To maximize simplicity and the potential for fundamental intuitions, we modeled the musculoskeletal system as a system of point masses (bones) and springs (muscles). We stretched a muscle-spring and observed the impact of this perturbation on the locations of all other muscles. Physically, to perturb a muscle, we displaced all bones connected to that muscle by the same amount and in the same direction, stretching the muscle, and we held these bones fixed at their new location. This process is also mathematically equivalent to simply altering the spring constant attributed to the particular muscle-spring. The system was then allowed to reach equilibrium. We fixed bones at the midline and around the periphery in space to prevent the system from drifting. To quantify the impact of perturbing this single muscle-spring, we defined the movement of the *i*th node as follows:

$$m_{i}\frac{d^{2}{\vec{r}}_{i}}{dt^{2}}=\sum_{j\neq i\in V}^{}[S_{ij}A_{ij}{\vec{l}}_{ij}(x_{ij}-\|{\vec{l}}_{ij}\|)]-\beta \frac{d{\vec{r}}_{i}}{dt},$$

where l_ij_ is the displacement between nodes i and j, x_ij_ is the unperturbed distance between nodes i and j, m is the mass of the node (which we have set equal to unity for all nodes in the network), β = 1 is a damping coefficient, r_i_ is the position of the *i*th node, A is the weighted adjacency matrix of the bone-centric graph, and S_ij_ represents the sum of all spring strengths of the muscles that nodes i and j are both connected to. To normalize muscles’ restoring force on nodes, we let the spring strength of a muscle q ∝ 1/(k − 1). Here, we have set all bones to have equal weight and all muscles to have equal spring constant, which is a simplification of the actual physical anatomy. For a discussion of how to account for additional physical properties, such as bone weight and muscle strength, and supplementary results using these properties, see S5 Text. Moreover, sample trajectories that provide an intuition for the dynamics of our model have been included in the Supporting information (S8 Fig).

To measure the potential functional role of each muscle in the network, we stretched a muscle hyperedge and measured the impact of the perturbation on the rest of the network. Rather than perturbing the network in some arbitrary three-dimensional direction, we extended the scope of our simulation into a fourth dimension. When perturbing a muscle, we displaced all of the nodes (bones) contained in that muscle hyperedge by a constant vector in the fourth dimension and held them with this displacement (Fig 1d). The perturbation then rippled through the network of springs in response. We sequentially stretched each muscle hyperedge and defined the impact score of this perturbation to be the total distance moved by all nodes in the musculoskeletal network from their original positions. The displacement value is the summed displacement over all time points, from perturbation onset to an appropriate cutoff for equilibration time. Here, we solved for the equilibrium of the system by allowing dynamics to equalize over a sufficient period of time. Note that the equilibrium can also be solved for using a steady-state, nondynamic approach; we chose to use dynamics in this instance to more broadly support future applications.

### Impact score deviation

For each muscle, we calculated an index that quantifies how much the impact score of that muscle deviates from expected, given its hyperedge degree; we call this index “impact deviation”. We begin by constructing a null model that dictates the expected impact under a set of statistical assumptions. In the current study, we used several different null models with differing sets of assumptions, which we detail in later sections. Impact deviation was computed as follows: we calculated the mean, standard deviation, and 95% confidence intervals (CIs) for each of the null hypergraph degree categories from an ensemble of 100 null hypergraphs. The distance from a given muscle to the mean ± 95% CI (whichever is closest) was calculated and divided by the standard deviation of that null hypergraph degree distribution. In this way, we calculated deviation from the expected value, in standard deviations (similar to a z-score). Table 1 contains the muscles that lie outside the 95% CI of deviation ratios, relative to their hyperedge degree. Muscles can be naturally grouped according to the homunculus, a coarse one-dimensional representation of how the control areas of muscles group onto the motor cortex. For a given homunculus group, we calculated the deviation ratio as the number of muscles with positive deviation divided by the total number of muscles in the group (Table 2).

**Table 1:** Muscles with greater and lesser impact than expected in a hypergraph null model. The muscles on the left side have less impact than expected, given their hyperedge degree: their impacts are more than 1.96 standard deviations below the mean, indicating that they lie outside the 95% confidence interval of the distribution. The muscles on the right side have more impact than expected given their hyperedge degree: their impacts are more than 1.96 standard deviations above the mean, ordered from most to least extreme. This table shows the muscles that had the greatest positive and greatest negative difference in impact, relative to degree-matched controls.

Rank order	Less impact than expected		More impact than expected	
Number	Hyperedge	Muscle name	Hyperedge	Muscle name
1	137	Orbicularis oculi	20	Brachialis
2	145	Buccinator	22	Anconeus
3	148	Medial pterygoid	18	Coracobrachialis
4			12	Teres minor
5			11	Infraspinatus
6			14	Subscapularis
7			13	Teres major
8			10	Supraspinatus
8			16	Pectoralis major
9			32	Extensor carpi radialus longus
11			161	Piriformis
10			31	Brachioradialis

**Table 2:** Homunculus categories whose member muscles either all have more impact than expected or all have less impact than expected, compared to null hypergraphs. Categories on the left are composed entirely of muscles with less impact than expected, compared to degree-matched controls. Categories on the right are composed entirely of muscles with more impact than expected, compared to degree-matched controls.

Rank order	Less impact than expected		More impact than expected	
Number	Hyperedge	Muscle name	Hyperedge	Muscle name
1	16	Brow	3	Knee
2	17	Eye muscles	4	Hip
3	18	Face muscles	6	Shoulder
4	19	Lip muscles	7	Elbow
5			8	Wrist
6			9	Hand
7			10	Little finger
8			21	Tongue

### Community detection

To understand both the function and control of the musculoskeletal system, we were interested in defining groups of densely interconnected muscles using a data-driven approach. We performed a type of community detection by maximizing a modularity quality function introduced by Newman [20]:

$$Q=\sum_{ij}B_{ij}-\gamma P_{ij}\delta \left(g_{i},g_{j}\right),$$

where P_ij_ is the expected weight of an edge in the Newman-Girvan null model, node i is assigned to community g_i_, node j is assigned to community g_j_, and δ is the Kronecker delta function. By maximizing Q, we obtained a partition of nodes (muscles) into communities such that nodes within the same community were more densely interconnected than expected in a network null model (Fig 1b, right).

Here, we also used a resolution parameter to tune the size and number of communities detected such that the number of communities detected matched the number of groups within the homunculus, for straightforward comparison. Specifically, we used a resolution parameter of γ = 4.3 to divide the muscle-centric matrix into 22 communities (see S8 Table). We began by redefining the original muscle-centric matrix B following Jutla et al. [21]; we let *k* = Σ_*i*_ *B*_*i*,*j*_, and then we applied a locally greedy, Louvain-like modularity maximization algorithm to the adjusted matrix $B\prime =B-\gamma \frac{k^{T}k}{\sum_{j}^{}k_{j}}$ [22].

The above method of community detection is nondeterministic [23]. That is, the same solution will not be reached on each individual run of the algorithm. Therefore, one must ensure that the community assignments used are a good representation of the network and not just a local maximum of the landscape. We therefore maximized the modularity quality function 100 times, obtaining 100 different community assignments. From this set of solutions, we identified a robust representative consensus community structure [24]. S1 Fig illustrates how the detected communities change as a function of the resolution parameter for the muscle-centric network.

### Network null models

We use rewired graphs as a null model against which to compare the empirical data. Specifically, we constructed a null hypergraph by rewiring muscles that are assigned the same category (Table 3, defined below) uniformly at random. In this way, muscles of the little finger will only be rewired within the little finger, and similarly for muscles in other categories. Importantly, this method also preserves the degree of each muscle as well as the degree distribution of the entire hypergraph.

**Table 3:** Homunculus categories and their associated identification numbers.

Category ID	Category name
1	Toes
2	Ankle
3	Knee
4	Hip
5	Trunk
6	Shoulder
7	Elbow
8	Wrist
9	Hand
10	Little finger
11	Ring finger
12	Middle finger
13	Index finger
14	Thumb
15	Neck
16	Brow
17	Eyelid and eyeball
18	Face
19	Lips
20	Jaw
21	Tongue
22	Swallowing

Categories were assigned to muscles such that the overall topology of the musculoskeletal system was grossly preserved, and changes were spatially localized. Specifically, we partitioned the muscles into communities of roughly size 3, such that each muscle was grouped with the two muscles that are most topologically related. We then permuted only within these small groups. This is a data-driven way of altering connections only within very small groups of related muscles.

To partition muscles into communities, we took a greedy approach to modularity maximization, similar to prior work [25]. Specifically, we maximized the modularity of the system, such that the change in modularity to move node n from community c′ to community c is given by

$$dQ_{nc}=H_{nc}-H_{nc^{\prime }}+{B\prime }_{nn}-V_{cc^{\prime }}$$

Here, H is the node-to-module degree matrix, B′ is the adjusted muscle-centric matrix, and V is a penalty term to ensure communities will be small and of roughly equal size. Specifically,

$$H_{nc}=\sum_{i=1}^{N}{B\prime }_{nm}\delta_{c_{j}m},$$

where N is the total number of nodes in the system, c_j_ is an indicator variable encoding the community assignment of node j, and δ is the Kronecker delta function. Furthermore,

$$V_{cc^{\prime }}={\left(\sum_{j=1}^{N}\delta_{c_{j}c^{\prime }}-\frac{N}{K}\right)}^{2},$$

where K indicates the total number of communities. This term penalizes determining a set of communities that are highly unequal in size.

### Multidimensional scaling

To conduct multidimensional scaling (MDS) on the muscle-centric network, the weighted muscle-centric adjacency matrix was simplified to a binary matrix (all nonzero elements set equal to 1). From this data, a distance matrix D was constructed, the elements D_ij_ of which are equal to the length of the shortest path between muscles i and j, or are equal to 0 if no path exists. MDS is then applied to this distance matrix to yield its first principal component using the MATLAB function, cmdscale.m. To construct the binary matrix, a threshold of 0 was set, and all values above that threshold were converted to 1. However, to make analysis robust to this choice, we explored a range of threshold values to verify that results are invariant with respect to threshold. The upper bound of the threshold range was established by determining the maximal value that would maintain a fully connected matrix; otherwise, the distance matrix D would have entries of infinite weight. In our case, this value was 0.0556 × max(B′). Within this range of thresholds (i.e., for all thresholds resulting in fully connected matrices), results were qualitatively consistent. As a supplementary analysis, we also employed a method of constructing a distance matrix from a weighted adjacency matrix in order to preclude thresholding (S5 Fig), and we again observed qualitatively consistent results.

### Muscle injury data

We calculated the correlation between impact score and muscle injury recovery times. Injury recovery times were collected from the sports medicine literature and included injury to the triceps brachii and shoulder muscles [26]; thumb muscles [27]; latissimus dorsi and teres major [28]; biceps brachii [29]; ankle muscles [30]; neck muscles [31]; jaw muscles [32]; hip muscles [33]; eye/eyelid muscles [34]; and muscles of the knee [35], elbow [36], and wrist/hand [37]. The recovery times and associated citations, listed in Table 4, are average recovery times gathered from population studies. If the literature reported a range of different severity levels and associated recovery times for a particular injury, the least severe level was selected. If the injury was reported for a group of muscles rather than a single muscle, the impact score deviation for that group was averaged together. Data points for muscle groups were weighted according to the number of muscles in that group for the purpose of the linear fit. The fit was produced using the MATLAB function, fitlm.m, with option “Robust” set to “on.” Robust regression is a method of regression designed to be less sensitive to outliers within the data, in which outliers are down-weighted in the regression model.

**Table 4:** Muscle injury recovery data.

Muscle	Weeks of recovery	Source
Triceps brachii	4	Bateman (1962)
Thumb muscles	4	Rettig (2004)
Latissimus dorsi	12	Nagda (2011)
Biceps brachii	12	Zafra (2009)
Ankle	2	McCollum (2012)
Neck	0.14	Torg (1982)
Jaw	0	Beachy (2004)
Shoulder	2	Bateman (1962)
Teres major	12	Nagda (2011)
Hip	12	Niemuth (2005)
Eye/eyelid	1.4	Leivo (2015)
Knee	8	Ekstrand (1982)
Elbow	8	Fleisig (2012)
Wrist/hand	1.4	Logan (2004)

### Somatotopic representation area data

We calculated the correlation between impact score deviation and the area of somatotopic representation devoted to a particular muscle group. The areas of representation were collected from two separate sources [38,39]. The volumes and associated citations are listed in Table 5. In both studies, subjects were asked to articulate a joint repetitively, and the volumes of the areas of primary motor cortex that underwent the greatest change in BOLD signal were recorded. We then calculated the correlation coefficient between cortical volumes and the mean impact of all muscles associated with that joint, as determined by the Hosford Muscle tables. We found a significant linear correlation between the two measures by using the MATLAB function, fitlm.m, with option “Robust” set to “on.”

**Table 5:** Primary motor cortex somatotopic representation volume sizes and sources.

Muscle	Volume (mm^3^)	Reference
Thumb	1,390	Indovina (2000)
Index	1,000	Indovina (2000)
Middle	650	Indovina (2000)
Hand	5,566	Alkadhi (2002)
Fingers	2,972	Alkadhi (2002)
Wrist	4,409	Alkadhi (2002)
Elbow	2,267	Alkadhi (2002)

## Results

### Structure of the human musculoskeletal network

**Fig 2:**
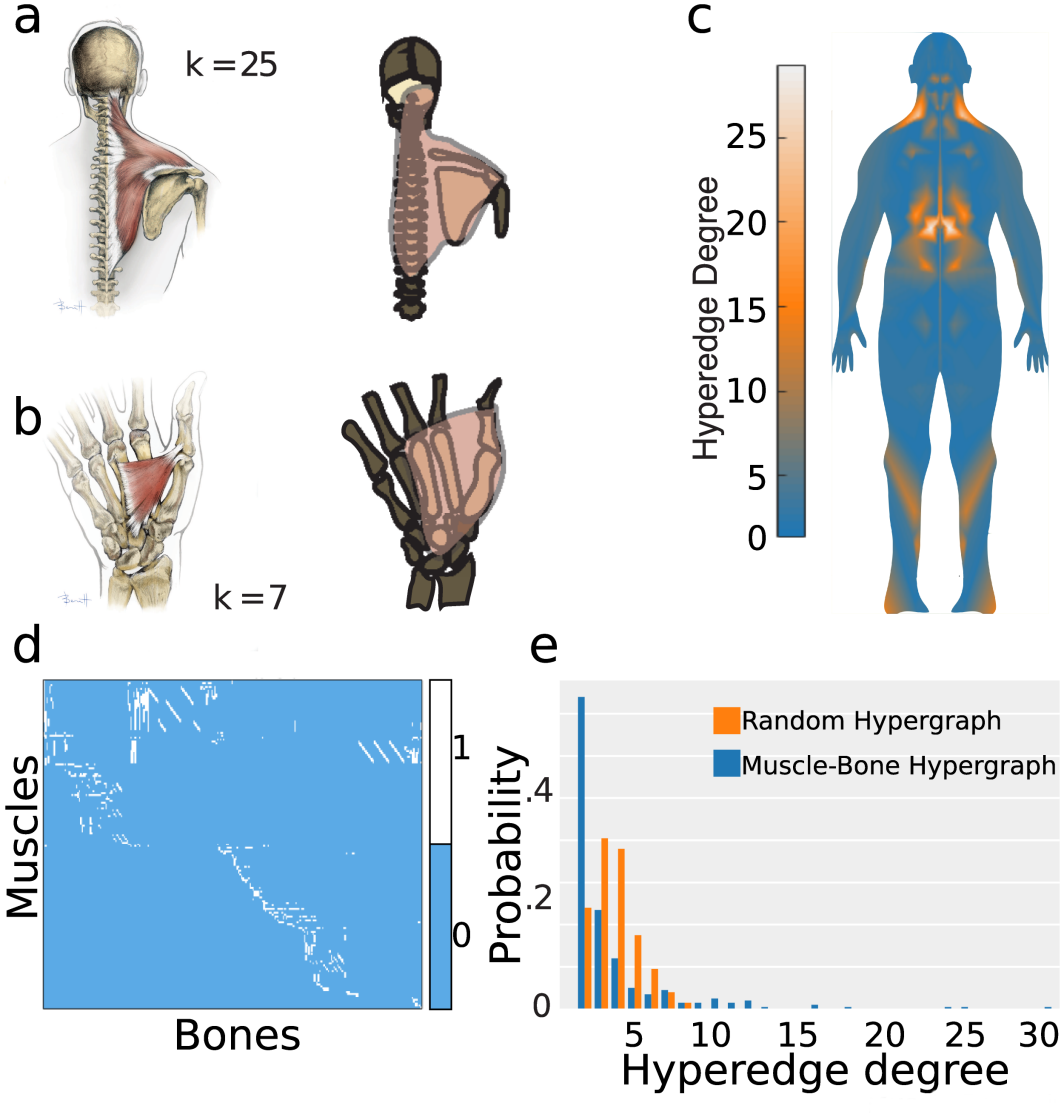
Hypergraph structure. (a) Left: Anatomical drawing highlighting the trapezius. Right: Transformation of the trapezius into a hyperedge (red; degree k = 25), linking 25 nodes (bones) across the head, shoulder, and spine. (b) Adductor pollicis muscle linking 7 bones in the hand. (c) Spatial projection of the hyperedge degree distribution onto the human body. High-degree hyperedges are most heavily concentrated at the core. (d) The musculoskeletal network displayed as a bipartite matrix (1 = connected, 0 otherwise). (e) The hyperedge degree distribution for the musculoskeletal hypergraph, which is significantly different than that expected in a random hypergraph. *Data available for (e) at DOI*:10.5281/zenodo.1069104.

To examine the structural interconnections of the human musculoskeletal system, we used a hypergraph approach. Drawing from recent advances in network science [5], we examined the musculoskeletal system as a network in which bones (network nodes) are connected to one another by muscles (network hyperedges). A hyperedge is an object that connects multiple nodes; muscles link multiple bones via origin and insertion points. The degree, k, of a hyperedge is equal to the number of nodes it connects; thus, the degree of a muscle is the number of bones it contacts. For instance, the trapezius is a high-degree hyperedge that links 25 bones throughout the shoulder blade and spine; conversely, the adductor pollicis is a low-degree hyperedge that links 7 bones in the hand (Fig 2a and 2b). A collection of hyperedges (muscles) that share nodes (bones) is referred to as a hypergraph: a graph H = (V, E) with N nodes and M hyperedges, where V = {v_1_,···, v_N_} is the set of nodes and E = {e_1_,···, e_M_} is the set of hyperedges.

The representation of the human musculoskeletal system as a hypergraph facilitates a quantitative assessment of its structure (Fig 2c). We observed that the distribution of hyperedge degree is heavy-tailed: most muscles link 2 bones, and a few muscles link many bones (Fig 2d and 2e). The skew of the degree distribution differs significantly from that of random networks (two-sample Kolmogorov-Smirnov test, KS = 0.37, *p* < 0.0001, see Materials and methods) [5], indicating the presence of muscles of unexpectedly low and high degree (Fig 2e).

### Function of the human musculoskeletal network

To probe the functional role of muscles within the musculoskeletal network, we employed a simplified model of the musculoskeletal system and probed whether the model could generate useful clinical correlates. We implemented a physical model in which bones form the core scaffolding of the body, while muscles fasten this structure together. Each node (bone) is represented as a mass, whose spatial location and movement are physically constrained by the hyperedges (muscles) to which it is connected. Specifically, bones are points located at their center of mass, derived from anatomy texts [19], and muscles are springs (damped harmonic oscillators) connecting these points [40,41]; for a hyperedge of degree k, we created k(k − 1)/2 springs linking the k nodes. That is, for a muscle connecting k bones, we placed springs such that each of the k muscles had a direct spring connection to each of the other k − 1 bones.

**Fig 3:**
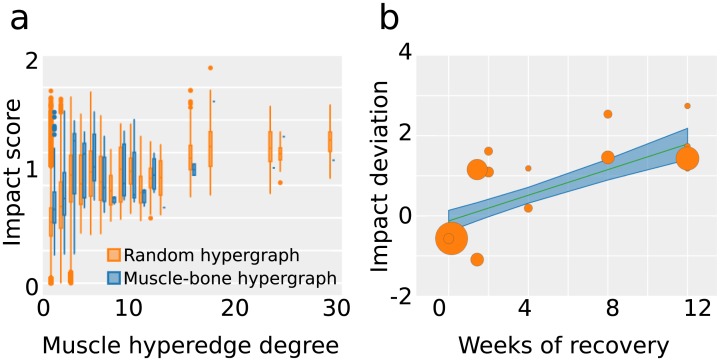
Probing musculoskeletal function. (a) The impact score plotted as a function of the hyperedge degree for a null hypergraph model and the observed musculoskeletal hypergraph. (b) Impact score deviation correlates with muscle recovery time following injury to muscles or muscle groups (F(1,12) = 37.3, R^2^ = 0.757, *p* < 0.0001). Shaded areas indicate 95% confidence intervals, and data points are scaled according to the number of muscles included. The plot is numbered as follows, corresponding to Table 4: triceps (1), thumb (2), latissimus dorsi (3), biceps brachii (4), ankle (5), neck (6), jaw (7), shoulder (8), teres major (9), hip (10), eye muscles (11), knee (12), elbow (13), wrist/hand (14). *Data available at DOI*:10.5281/zenodo.1069104.

Next, we perturbed each of 270 muscles in the body and calculated their impact score on the network (see Materials and methods and Fig 1c and 1d). As a muscle is physically displaced, it causes a rippling displacement of other muscles throughout the network. The impact score of a muscle is the mean displacement of all bones (and indirectly, muscles) resulting from its initial displacement. We observed a significant positive correlation between muscle degree and impact score (F(1,268) = 23.3, R^2^ = 0.45, *p* < 0.00001; Fig 3a), suggesting that hyperedge structure dictates the functional role of muscles in the musculoskeletal network. Muscles with a larger number of insertion and origin points have a greater impact on the musculoskeletal system when perturbed than muscles with few insertion and origin points [42]. We can gain further insights into the results of these analyses by explicitly studying the relation between impact score and statistical measures of the network’s topology. In S11 Fig, we show that the network function as measured by the impact score was significantly correlated with the average shortest path length. While the network statistics are static in nature, their functional interpretation is provided by the perturbative simulations of system dynamics.

To guide interpretation, it is critical to note that the impact score, while significantly correlated with muscle degree, is not perfectly predicted by it (Fig 3a). Instead, the local network structure surrounding a muscle also plays an important role in its functional impact and ability to recover. To better quantify the effect of this local network structure, we asked whether muscles existed that had significantly higher or significantly lower impact scores than expected in a null network. We defined a positive (negative) impact score deviation that measures the degree to which muscles are more (less) impactful than expected in a network null model (see Materials and methods). This calculation resulted in a metric that expresses the impact of a particular muscle, relative to muscles of identical hyperedge degree in the null model. In other words, this metric accounts for the complexity of a particular muscle (Table 1).

Is this mathematical model clinically relevant? Does the body respond differently to injuries to muscles with higher impact score than to muscles with lower impact score? To answer this question, we assessed the potential relationship between muscle impact and recovery time following injury. Specifically, we gathered data on athletic sports injuries and the time between the initial injury and return to sport. Critically, we observed that recovery times were strongly correlated with impact score deviations of the individual muscle or muscle group injured (F(1,12) = 37.3, R^2^ = 0.757, *p* < 0.0001; Fig 3b), suggesting that our mathematical model offers a useful clinical biomarker for the network’s response to damage. We note that it is important to consider the fact that recovery might be slower in a person who is requiring maximal effort in a performance sport, compared to an individual who is seeking only to function in day-to-day life. In order to generalize our findings to the entire population, we therefore also examined recovery time data collected from nonathletes, and we present these complementary results in the Supporting information (S6 Text).

Finally, to provide intuition regarding how focal injury can produce distant effects potentially slowing recovery, we calculated the impact of the ankle muscles and determined which other muscles were most impacted. That is, for each individual ankle muscle, we calculated the impact on each of the remaining 264 non-ankle muscles and then averaged this over all ankle muscles. Out of the 264 non-ankle muscles, the single muscle that is most impacted by the perturbation of ankle muscles is the biceps femoris of the hip, and the second most impacted is the vastus lateralis of the knee. Additionally, the muscle most impacted by perturbation to hip muscles is the soleus.

### Control of the human musculoskeletal network

**Fig 4:**
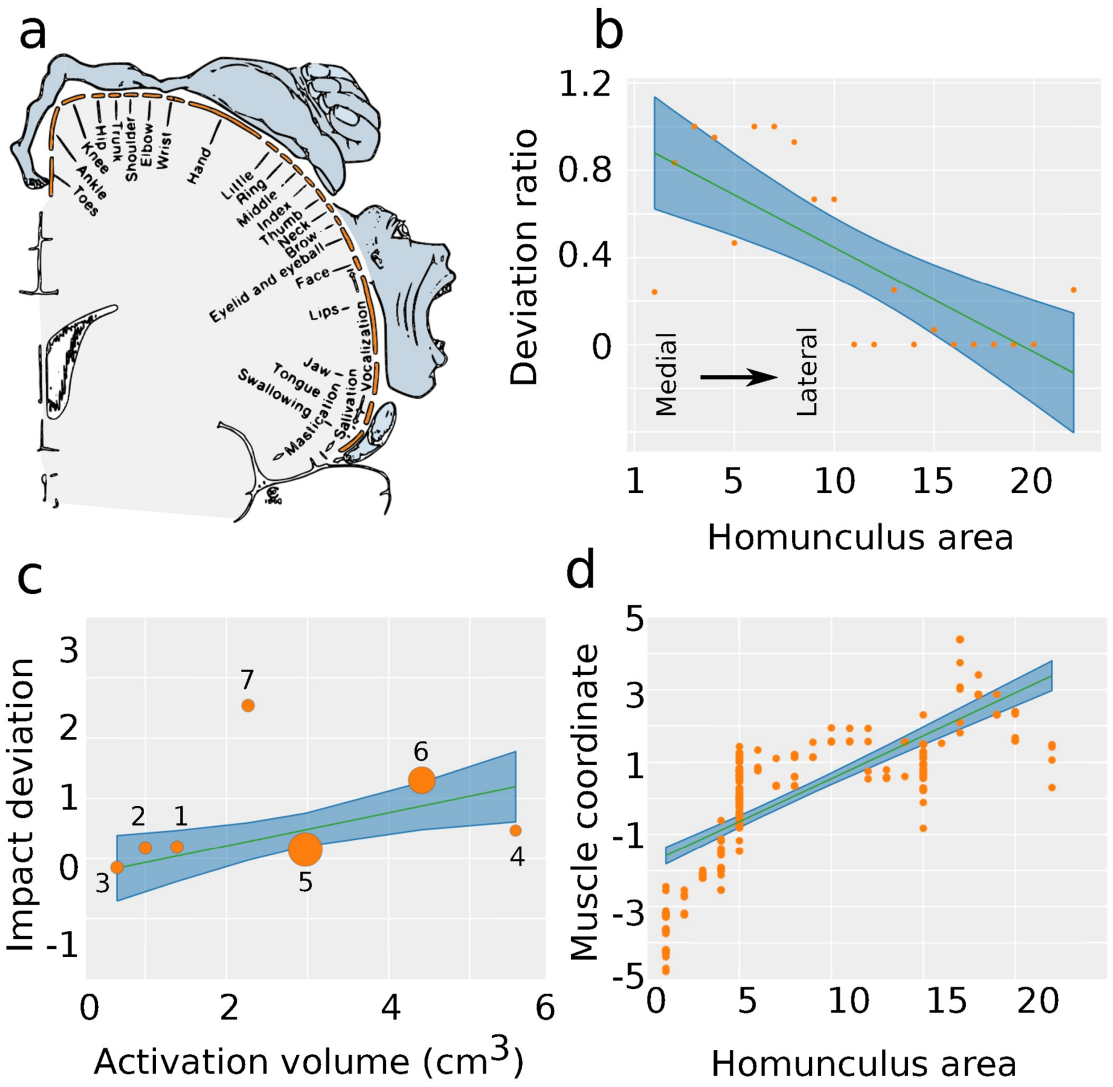
Probing musculoskeletal control. (a) The primary motor cortex homunculus as constructed by Penfield. (b) Deviation ratio correlates significantly with homuncular topology (F(1,19) = 21.3, R^2^ = 0.52, *p* < 0.001), decreasing from medial (area 0) to lateral (area 22). (c) Impact score deviation significantly correlates with motor strip activation volume (F(1,5) = 14.4, R^2^ = 0.743, *p* = 0.012). Data points are sized according to the number of muscles required for the particular movement. The plot is numbered as follows, corresponding to Table 5: thumb (1), index finger (2), middle finger (3), hand (4), all fingers (5), wrist (6), elbow (7). (d) Correlation between the spatial ordering of Penfield’s homunculus categories and the linear muscle coordinate from a multidimensional scaling analysis (F(1,268) = 316, R^2^ = 0.54, *p* < 0.0001). *Data available at DOI*:10.5281/zenodo.1069104.

What is the relationship between the functional impact of a muscle on the body and the neural architecture that affects control? Here, we interrogate the relationship between the musculoskeletal system and the primary motor cortex. We examined the cerebral cortical representation map area devoted to muscles with low versus high impact by drawing on the anatomy of the motor strip represented in the motor homunculus [43] (Fig 4a), a coarse one-dimensional representation of the body in the brain [44]. We observed that homunculus areas differentially control muscles with positive versus negative impact deviation scores (Table 2). Moreover, we found that homunculus areas controlling only positively (negatively) deviating muscles tend to be located medially (laterally) on the motor strip, suggesting the presence of a topological organization of a muscle’s expected impact in neural tissue. To probe this pattern more deeply, for each homunculus area, we calculated a deviation ratio as the percent of muscles that positively deviated from the expected impact score (i.e., a value of 1 for brow, eye, face and a value of 0 for knee, hip, shoulder; see Table 2). We found that the deviation ratio was significantly correlated with the topological location on the motor strip (F(1,19) = 21.3, R^2^ = 0.52, *p* < 0.001; Fig 4b).

As a stricter test of this relationship between a muscle’s impact on the network and neural architecture, we collated data for the physical volumes of functional MRI-based activation on the motor strip that are devoted to individual movements (e.g., finger flexion or eye blinks). Activation volumes are defined as voxels that become activated (defined by blood-oxygen-level-dependent signal) during movement [38,39]. Critically, we found that the functional activation volume independently predicts the impact score deviation of muscles (Fig 4c, F(1,5) = 14.4, *p* = 0.012, R^2^ = 0.743), consistent with the intuition that the brain would devote more real estate in gray matter to the control of muscles that are more impactful than expected in a null model. Again, impact deviation is a metric that accounts for the hyperedge degree of a particular muscle and is relative to the impact of muscles with identical hyperedge degree in the null model. Thus, the impact deviation measures the local network topology beyond simply the immediate connections of the muscle in question.

As a final test of this relationship, we asked whether the neural control strategy embodied by the motor strip is optimally mapped to muscle groups. We constructed a muscle-centric graph by connecting two muscles if they touch on the same bone (Fig 1c, left). We observed the presence of groups of muscles that were densely interconnected with one another, sharing common bones. We extracted these groups using a clustering technique designed for networks [45,46], which provides a data-driven partition of muscles into communities (Fig 1b, right). To compare the community structure present in the muscle network to the architecture of the neural control system, we considered each of the 22 categories in the motor homunculus [18] as a distinct neural community and compared these brain-based community assignments with the community assignments obtained from a data-driven partition of the muscle network. Using the Rand coefficient [47], we found that the community assignments from both homunculus and muscle network were statistically similar (z_Rand_ > 10), indicating a correspondence between the modular organization of the musculoskeletal system and the structure of the homunculus. For example, the triceps brachii and the biceps brachii belong to the same homuncular category, and we found that they also belong to the same topological muscle network community.

Next, because the homunculus has a linear topological organization, we asked whether the order of communities within the homunculus (Table 3) was similar to a data-driven ordering of the muscle groups in the body, as determined by MDS [48]. From the muscle-centric network (Fig 1b), we derived a distance matrix that encodes the smallest number of bones that must be traversed to travel from one muscle to another. An MDS of this distance matrix revealed a one-dimensional linear coordinate for each muscle, such that topologically close muscles were close together and topologically distant muscles were far apart. We observed that each muscle’s linear coordinate is significantly correlated with its homunculus category (Fig 4d, F(1,268) = 316, *p* < 0.0001, R^2^ = 0.54), indicating an efficient mapping between the neural representation of the muscle system and the network topology of the muscle system in the body.

Our results from Fig 4d demonstrate a correspondence between the topology of the homunculus and a data-driven ordering of muscles obtained by considering the topological distances between them. This result could be interpreted in one of two ways: one reasonable hypothesis is that because most connections in the musculoskeletal network are short range, the finding is primarily driven by short-range connections. A second reasonable hypothesis is that while short-range connections are the most prevalent, long-range connections form important intramodular links that help determine the organization of the network. To arbitrate between these two hypotheses, we considered two variations of our MDS experiment: one including only connections shorter than the mean connection length and the other including only connections longer than the mean connection length. We found that the data-driven ordering derived from only short and only long connections both led to significant correlations with the homuncular topology (F(1,268) = 24.9, R^2^ = 0.085, *p* < 0.0001 and F(1,268) = 5, R^2^ = 0.018, *p* = 0.026, respectively). Notably, including both long and short connections leads to a stronger correlation with homuncular topology than considering either independently, suggesting a dependence on connections of all lengths. It would be interesting in the future to test the degree to which this network-to-network map is altered in individuals with motor deficits or changes following stroke.

## Discussion

### Structure of the human musculoskeletal network

By representing the complex interconnectivity of the musculoskeletal system as a network of bones (represented by nodes) and muscles (represented by hyperedges), we gained valuable insight into the organization of the human body. The study of anatomical networks using similar methods is becoming more common in the fields of evolutionary and developmental biology [10]. However, the approach has generally been applied only to individual parts of the body—including the arm [49], the head [11], and the spine [12]—thereby offering insights into how that part of the organism evolved [50,51]. Moreover, even when full body musculature [13] and the neuromusculoskeletal [14] system more generally have been modeled, some quantitative claims can remain elusive, in large part due to the lack of a mathematical language in which to discuss the complexity of the interconnection patterns. In this study, we offer an explicit and parsimonious representation of the complete musculoskeletal system as a graph of nodes and edges, and this representation allowed us to precisely characterize the network in its entirety.

When modeling a system as a network, it is important to begin the ensuing investigation by characterizing a few key architectural properties. One particularly fundamental measure of a network’s structure is its degree distribution [52], which describes the heterogeneity of a node’s connectivity to its neighbors in a manner that can provide insight into how the system formed [7]. We observed that the degree distribution of the musculoskeletal system is significantly different from that expected in a null graph (Fig 2e), displaying fewer high-degree nodes and an overabundance of low-degree nodes. The discrepancy between real and null model graphs is consistent with the fact that the human musculoskeletal system develops in the context of physical and functional constraints that together drive its decidedly nonrandom architecture [53]. The degree distribution of this network displays a peak at approximately degree two, that is then followed by a relatively heavy tail of high-degree nodes. The latter feature is commonly observed in many types of real-world networks [54], whose hubs may be costly to develop, maintain, and use [55,56] but play critical roles in system robustness, enabling swift responses [55], buffering environmental variation [57], and facilitating survival and reproduction [58]. The former feature—the distribution’s peak—is consistent with the intuition that most muscles within the musculoskeletal system connect with only two bones, primarily for the function of simple flexion or extension at a joint. By contrast, there are only a few muscles that require a high degree to support highly complex movements, such as maintaining the alignment and angle of the spinal column by managing the movement of many bones simultaneously. These expected findings provide important validation of the model as well as offer a useful visualization of the musculoskeletal system.

The musculoskeletal network is characterized by a particularly interesting property that distinguishes it from several other real-world networks: the fact that it is embedded into three-dimensional space [59]. This property is not observed in semantic networks [60] or the World Wide Web [61], which encode relationships between words, concepts, or documents in some abstract (and very likely non-euclidean) geometry. In contrast, the musculoskeletal system composes a volume, with nodes having specific coordinates and edges representing physically extended tissues. To better understand the physical nature of the musculoskeletal network, we examined the anatomical locations of muscles with varying degrees (Fig 2c). We observed that muscle hubs occur predominantly in the torso, providing dense structural interconnectivity that can stabilize the body’s core and prevent injury [62]. Specifically, high-degree muscles cluster about the body’s midline, close to the spine, and around the pelvic and shoulder girdle, consistent with the notion that both agility and stability of these areas requires an ensemble of muscles with differing geometries and tissue properties [63]. Indeed, muscles at these locations must support not only flexion and extension but also abduction, adduction, and both internal and external rotation.

It is important to note that significant variation exists within the musculoskeletal system across individuals, and not all anatomical atlases agree on the most representative set of insertion and origin points. The results presented here reflect how the musculoskeletal system was presented in the text from which it was constructed [19] and therefore provide only one possible network representation of the musculoskeletal system. To assess the reliability of our results across reasonable variation of the musculoskeletal configuration, we created a second musculoskeletal network from an alternate atlas [64]. Using this second atlas, we observed consistent results, and we report these complementary analyses in S3 Text.

It is also important to note that we mapped the first atlas [19] into a musculoskeletal graph composed of both bony and non-bony nodes. This choice equates the structural roles of bones and certain tendons and ligaments, which is admittedly a simplification of the biology. One justification for this simplification is that non-bony structures frequently serve as critical attachment points of muscles (i.e., the plantar fascia of the foot). Thus, it is reasonable to separate the musculoskeletal network into the two categories of muscles and structures that serve as muscular attachment points, as we did here. Nonetheless, this second category is quite heterogeneous in composition, and in future work, one could also consider constructing a multilayer graph, with a separate layer accounting for each type of muscular attachment structure. To confirm that our findings and interpretations are not significantly altered by the presence of non-bony muscular attachment points, we removed such points in an alternative atlas and observe that our main findings still hold (see S3 Text).

### Function of the human musculoskeletal network

To better understand the functional role of a single muscle within the interconnected musculoskeletal system, we implemented a physics-based model of the network’s impulse response properties by encoding the bones as point masses and the muscles as springs [65]. Significantly, this highly simplified model of the musculoskeletal system is able to identify important functional features. While muscles of high degree also tended to have a large impact on the network’s response (Fig 3a), there were several notable deviations from this trend (Table 1).

The muscle noted to have the least impact relative to that expected is the orbicularis oculi, the muscle used for controlling movement of the eyelid. This muscle is small and relatively isolated in the body, originating and inserting on bones of the skull. The face muscles in general form a tight and isolated community, with few connections reaching outside that community. These factors likely contribute to the low impact of this muscle, and an analogous argument could be made for the remaining two muscles with less impact than expected, which are also muscles of the face.

The muscles with more impact than expected are more numerous but almost entirely located in the upper limb or upper limb girdle. The extensor carpi radialis longus, anconeus, brachioradialis, and brachialis muscles are all intrinsic arm muscles, the latter three acting at the elbow. All of these muscles may have higher impact than expected in a null model because they can either directly or indirectly affect the movement of the many bones of the wrist and hand. The observed high impact of these muscles could be a result of the fact that they control the movement of a limb, and at the end of the limb are many bones whose movement depends directly on these muscles. The remainder of the high-impact muscles, with the exception of the piriformis, all attach the upper limb to the axial skeleton. These muscles are the coracobrachialis, infraspinatus, supraspinatus, subscapularis, teres minor, teres major, and pectoralis major muscles. These muscles, like the previous four, have the property that they control the movement of an entire limb, which likely contributes to their impact. Unlike the previous group, these muscles also connect to the axial skeleton, which may also add to their impact. Many of these muscles originate on bones of the shoulder girdle and have the potential to affect all other shoulder girdle muscles, and potentially all bones connected to those muscles. This same dynamic likely exists in the lower limb, which is reflected by the presence of the piriformis muscle of the pelvic girdle. An in-depth discussion of how local network structure and muscle configuration may interact with impact deviation is presented in S7 Text. In addition to our work presented in the Supporting information, further insight may be gained into the properties of these outliers by performing experiments to closely examine the bones that are impacted most by each of these muscles.

While the network representation of a system can provide basic physical intuitions due to its parsimony and simplicity, it also remains agnostic to many details of the system’s architecture and function. It is a perennial question whether these first-principles models of complex systems can provide accurate predictions of real-world outcomes. We addressed this question by studying the relationship between the impact score of a muscle and the amount of time it takes for a person to recover from an injury. We quantified time of recovery by summing (i) the time to recover from the primary disability of the initial muscle injury and (ii) the time to recover from any secondary disabilities resulting from altered usage of other muscles in the network, due to the initial muscle injury [66]. We found that the deviation from the expected impact score in a null network correlated significantly with time of recovery (Fig 3b), supporting the notion that focal injury can have extended impacts on the body due to the inherently interconnected nature of the musculoskeletal system.

Indeed, muscular changes in one part of the body are known to affect other muscle groups. For example, strengthening hip muscles can lead to improved knee function following knee replacement [67]. Alteration of muscular function in the ankle following sprains can cause altered hip muscle function [68,69], a result replicated by our model (which found the biceps femoris and vastus lateralis were most impacted by ankle injury), and injury to limb muscles can lead to secondary injury of the diaphragm [70]. Our model offers a mathematically principled way in which to predict which muscles are more likely to have such a secondary impact on the larger musculoskeletal system and which muscles are at risk for secondary injury, given primary injury at a specific muscle site. It would be interesting in the future to test whether these predictions could inform beneficial adjustments to clinical interventions by explicitly taking the risk of secondary injury to particular muscles into account. Previously, prevention of secondary muscle injury has been largely relegated to cryotherapy [71,72] and has yet to be motivated by such a mechanistic model. Finally, an important question to ask is how this musculoskeletal configuration is evolutionarily advantageous and how evolutionary pressures may have optimized muscle impacts. Intuitively, one might expect that evolutionary pressures drive muscle impact down, perhaps by increasing muscular redundancy. A thorough investigation of the evolutionary advantages of the musculoskeletal network topology would be an interesting topic for future work.

### Control of the human musculoskeletal system

Given the complexity of the musculoskeletal network and its critical role in human survival, it is natural to ask questions about how that network is controlled by the human brain. Indeed, the study of motor control has a long and illustrious history [73], which has provided important insights into how the brain is able to successfully and precisely make voluntary movements despite challenges such as redundancies, noise [74], delays in sensory feedback [75], environmental uncertainty [76], neuromuscular nonlinearity [77], and nonstationarity [78]. Here, we took a distinct yet complementary approach and asked how the topology of the musculoskeletal network may be mapped onto the topology of the motor strip within the cortex. We began by noting that the impact deviation of a muscle is positively correlated with the size of the cortical volume devoted to its control (Fig 4c). One interpretation of this relationship is that those muscles with greater impact than expected in a null model by their immediate connections tend to control more complex movements and therefore necessitate a larger number of neurons to manage those movements [79]. A second interpretation builds on an evolutionary argument that muscles with more impact need a greater redundancy in their control systems [80], and this redundancy takes the form of a greater cortical area.

Local cortical volumes aside [81], one might also wish to understand to what degree the larger-scale organization of the musculoskeletal network reflects the organization of the motor strip that controls it. Building on the recent application of community detection techniques to the study of skull anatomy [11,82,83], we reported the modular organization of the muscle network: groups of muscles in which the muscles in one group are more likely to connect to one another than to muscles in other groups. More intriguingly, we observed that muscle communities closely mimic the known muscle grouping of the motor strip (Fig 1b, right): muscles that tend to connect to the same bones as each other also tend to be controlled by the same portion of the motor strip. Furthermore, a natural linear ordering of muscle communities—such that communities are placed close to one another on a line if they share network connections—mimics the order of control in the motor strip (Fig 4d). These results extend important prior work suggesting that the one-dimensional organization of the motor strip is related to both the structural and functional organization of the musculoskeletal network [84,85]. In fact, the results more specifically offer a network-level definition for optimal network control: the consistency of the linear map from musculoskeletal communities to motor strip communities.

Finally, we interrogated the physical locations of the cortical control of impactful muscles. We observed that muscles with more impact than expected given a null graph tend to be controlled by medial points on the motor strip, while muscles with less impact than expected tend to be controlled by lateral points on the motor strip (Fig 4b). This spatial specificity indicates that the organization of the motor strip is constrained by the physical layout of the body as well as aspects of how muscles function. Previous studies have examined a general temporal correspondence between cortical activity and muscle activity during movement [86], but little is known about topological correspondence.

### Methodological considerations

The construction of a hypergraph from the human musculoskeletal system requires assumptions and simplifications that impact the flexibility of the current model. Most prominent is the reduction of the system into two categories: muscles and bones. These categories hold no additional information and therefore do not account for features of a muscle’s or bone’s internal architecture. This simplification introduces several limitations to the perturbative model, including the capability of modeling the functional architecture of complex muscles, or those with the ability to independently contract a subset of fibers. For example, the two-headed biceps brachii has an origin both on the scapula and supraglenoid tubercle, and it is possible to contract the fibers of one head separately from the fibers of the other head. Future work could extend our modeling framework to represent this complex functional architecture. Furthermore, nonmuscular soft tissue structures essential to the musculoskeletal system cannot be explicitly accounted for. These structures, including tendons and ligaments, can either be (1) encoded as bones, as in the main text network, or (2) excluded from the network, as in the supplement; neither option is completely anatomically accurate.

In the case of bones, the model is unable to account for bone–bone interactions (joints). The majority of muscles act at joints, and the exclusion of joints obfuscates the specific function of muscles. That is, the model accounts for the fact that muscles move bones but not how they move or in what direction. In the perturbative simulation, the lack of joint constraints allows bones to be placed at unnatural angles relative to adjacent bones. In addition, bones are modeled as point masses, which in the perturbative simulation may allow bones to undergo trajectories involving the passage through space that, in reality, is occupied by another bone. Future work could extend our modeling framework to account for these additional biophysical constraints.

Insights generated by this model are a result of the input data. As individual variation exists within the musculoskeletal system, it similarly exists in muscle impacts. We have made an effort to use two input datasets to justify our main findings, but these findings may not be generalizable to all healthy musculoskeletal configurations. Specifically, the degree of a muscle, subject to individual variation, is likely to affect the impact of that muscle. Exactly how normative individual variation in muscle degree is related to variation in predicted muscle impact is an important question that, nevertheless, is outside of the scope of the current study.

Lastly, the human musculoskeletal system is a complex and densely interconnected network. Neither muscles nor bones function as independent entities. As such, it is difficult to parse the function of a single muscle from effects due to surrounding muscles. Nonindependence of muscles can be partially eliminated by appropriate null model selection, and our results hold under a variety of choices. Nonetheless, the notion that muscles—and impact factors—are not truly independent should be considered when interpreting these results.

## Conclusion

In summary, here we developed a novel network-based representation of the musculoskeletal system, constructed a mathematical modeling framework to predict recovery, and validated that prediction with data acquired from athletic injuries. Moreover, we directly linked the network structure of the musculoskeletal system to the organization of cortical architecture, suggesting an evolutionary pressure for optimal network control of the body. We compared the structure, function, and control of the human musculoskeletal system to a null system in which small groups of closely related muscles are rewired with each other. Our results suggest that the structure, function, and control of the musculoskeletal system are emergent from the highly detailed, small-scale organization, and when this small-scale organization is destroyed, so are the emergent features. Our work directly motivates future studies to test whether faster recovery may be attained by not only focusing rehabilitation on the primary muscle injured but also directing efforts towards muscles that the primary muscle impacts. Furthermore, our work supports the development of a predictive framework to determine the extent of musculoskeletal repercussions from insults to the primary motor cortex. An important step in the network science of clinical medicine [87], our results inform the attenuation of secondary injury and the hastening of recovery.

## Supporting information

S1 Text Alternative null models. This text file details construction of the alternative null models.
(DOCX)

S2 Text Resolution of community detection. This file provides a description of the choice of community detection resolution parameter.
(DOCX)

S3 Text Alternative musculoskeletal network. This file provides a description of the alternative musculoskeletal network.
(DOCX)

S4 Text Bone dynamics resulting from muscle perturbation. (DOCX)

S5 Text Accounting for bone weights and muscle strengths. (DOCX)

S6 Text Nonathlete muscle recovery analysis. (DOCX)

S7 Text Local network structure and impact deviation. (DOCX)

S1 Table Muscles with greater and lesser impact than expected in randomly rewired hypergraphs. This null model required randomly rewiring muscles within the hypergraph, preserving degree. The muscles on the left side have less impact than expected, given their hyperedge degree: their impacts are more than 1.96 standard deviations below the mean, indicating that they lie outside the 95% CI of the distribution. The muscles on the right side have more impact than expected, given their hyperedge degree: their impacts are more than 1.96 standard deviations above the mean, ordered from most to least extreme. This table shows the muscles that had the greatest positive and greatest negative difference in impact, relative to degree-matched controls.
(XLSX)

S2 Table Homunculus categories, the member muscles of which either all have more impact than expected or all have less impact than expected, compared to randomly rewired hypergraphs. This null model required randomly rewiring muscles within the hypergraph, preserving degree. Categories on the left are composed entirely of muscles with less impact than expected, compared to degree-matched controls. Categories on the right are composed entirely of muscles with more impact than expected, compared to degree-matched controls.
(XLSX)

S3 Table Muscles with greater and lesser impact than expected in hypergraphs randomly rewired within their homunculus category. This null model required randomly rewiring muscles within their homunculus category, preserving degree. The muscles on the left side have less impact than expected, given their hyperedge degree: their impacts are more than 1.96 standard deviations below the mean, indicating that they lie outside the 95% CI of the distribution. The muscles on the right side have more impact than expected, given their hyperedge degree: their impacts are more than 1.96 standard deviations above the mean, ordered from most to least extreme. This table shows the muscles that had the greatest positive and greatest negative difference in impact, relative to degree-matched controls.
(XLSX)

S4 Table Homunculus categories, member muscles of which either all have more impact than expected or all have less impact than expected, compared to hypergraphs randomly rewired within their homunculus category. This null model required randomly rewiring muscles within their homunculus category, preserving degree. Categories on the left are composed entirely of muscles with less impact than expected, compared to degree-matched controls. Categories on the right are composed entirely of muscles with more impact than expected, compared to degree-matched controls.
(XLSX)

S5 Table Muscles with greater and lesser impact than expected in a random hypergraph. This null model required randomly assigning muscle–bone connections, only preserving overall degree and not individual muscle degree. The muscles on the left side have less impact than expected, given their hyperedge degree: their impacts are more than 1.96 standard deviations below the mean, indicating that they lie outside the 95% CI of the distribution. The muscles on the right side have more impact than expected, given their hyperedge degree: their impacts are more than 1.96 standard deviations above the mean and are ordered from most to least extreme.
(XLSX)

S6 Table Volumes of muscles of the leg sub-network. Here, we include the muscle name (column 1), the muscle volume (in cm^3^; column 2), and the reference from which the estimate was taken.
(XLSX)

S7 Table Masses of the bones of the leg sub-network. Here, we include the bone name (column 1), the bone mass (in g; column 2), and the reference from which the estimate was taken.
(XLSX)

S8 Table The assigned homunculus categories and data-driven community assignments of muscles. Also available at DOI:10.5281/zenodo.1069104.
(XLSX)

S9 Table The hypergraph of muscles and bones from the Hosford Muscle tables [18], used in the main text. Also available at DOI:10.5281/zenodo.1069104.
(XLSX)

S10 Table The hypergraph of muscles and bones from Grant’s atlas [64], used in the supplementary text. Also available at DOI:10.5281/zenodo.1069104.
(XLSX)

S1 Fig Community detection with differing resolution parameters. This figure illustrates how the selection of the resolution parameter during community detection will change the number and size of communities detected. As the resolution parameter is increased, the size of individual communities decreases, while the number of communities increases. (a-d) Community detection for the muscle-centric network, using γ values of 1, 2, 8, and 16, respectively. The final community structure for each γ is a consensus partition of 100 individual runs of the community detection algorithm.
(EPS)

S2 Fig Community detection with differing resolution parameters. This figure illustrates stability around the chosen tuning parameter of γ = 4.3. Here, we explore partitions generated from nearby resolution parameters γ = 4.2 and γ = 4.4. Visually, the three partitions appear to have similar structure. The two nearby partitions are also mathematically similar, with z-score of the Rand coefficient [47] z_Rand_(γ = 4.2, γ = 4.3) = 105, z_Rand_(γ = 4.3, γ = 4.4) = 110, and z_Rand_(γ = 4.2, γ = 4.4) = 105. The final community structure for each γ is a consensus partition of 100 individual runs of the community detection algorithm.
(EPS)

S3 Fig Visual comparison of null models. This figure illustrates the differences in the null bipartite graphs. (A) The original unpermuted muscle–bone bipartite graph. (B) The random null bipartite graph. (C) The randomly rewired bipartite graph. (D) The in-community randomly rewired bipartite graph used in the main text, which permutes topology locally while preserving global topology.
(EPS)

S4 Fig Main results as a function of the null model. Here, we show results using a random hypergraph model or a rewired (permuted) hypergraph model that does not maintain local connections. (A) The impact score plotted as a function of the hyperedge degree for random hypergraphs and the observed musculoskeletal hypergraph. (B) The impact score plotted as a function of the hyperedge degree for permuted hypergraphs and the observed musculoskeletal hypergraph. (C) Deviation ratio correlates significantly with homuncular category (F(1,19) = 6.67, *p* = 0.018, R^2^ = 0.26), decreasing from medial (area 0) to lateral (area 22) using a random hypergraph null model. (D) Deviation ratio correlates significantly with homuncular category (F(1,19) = 6.86, *p* = 0.017, R^2^ = 0.26), decreasing from medial (area 0) to lateral (area 22) using a permuted hypergraph null model. (E) Impact score deviation significantly correlates with motor strip activation area (F(1,5) = 13.4, *p* = 0.014, R^2^ = 0.72) using a random hypergraph null model. Data points are sized according to the number of muscles required for the particular movement. (F) Impact score deviation significantly correlates with motor strip activation area (F(1,5) = 13.7, *p* = 0.022, R^2^ = 0.73) using a permuted hypergraph null model. Data points are sized according to the number of muscles required for the particular movement. (G) Impact score deviation correlates with muscle recovery time following injury to muscles or muscle groups (F(1,11) = 64.5, *p* = 6.3 × 10^−6^, R^2^ = 0.85), using a random hypergraph null model. Data points are scaled according to the number of muscles included. (H) Impact score deviation correlates with muscle recovery time following injury to muscles or muscle groups (F(1,11) = 70.5, *p* < 0.0001, R^2^ = 0.86), more so than expected in a permutation-based hypergraph null model. Data points are scaled according to the number of muscles included. Data available at DOI:10.5281/zenodo.1069104.
(EPS)

S5 Fig Network topology and the homunculus. Linear muscle coordinates determined using multidimensional scaling without thresholding via a weighted distance matrix (calculated using distance_wei.m included in the Brain Connectivity Toolbox, https://sites.google.com/site/bctnet/). Without thresholding, a significant correlation also exists between linear muscle coordinate and homunculus area (F(1,268) = 303, *p* < 0.0001, R^2^ = 0.53). Data available at DOI:10.5281/zenodo.1069104.
(EPS)

S6 Fig Probing musculoskeletal function for an alternate network. (a) The impact score plotted as a function of the hyperedge degree for a null hypergraph model and the observed musculoskeletal hypergraph. (b) Impact score deviation correlates with muscle recovery time following injury to muscles or muscle groups (F(1,12) = 40.2, *p* < 0.0001, R^2^ = 0.77). Shaded areas indicate 95% CIs, and data points are scaled according to the number of muscles included. Data available at DOI:10.5281/zenodo.1069104.
(PNG)

S7 Fig Probing musculoskeletal control for an alternate network. (a) Deviation ratio is significantly correlated with homuncular topology (F(1,18) = 8.88, R^2^ = 0.33, *p* = 0.0080), decreasing from medial (area 0) to lateral (area 22) regions. (b) Impact score deviation is significantly correlated with motor strip activation area (F(1,5) = 23.4, R^2^ = 0.82, *p* = 0.005). Data points are sized according to the number of muscles required for the particular movement. Data available at DOI:10.5281/zenodo.1069104.
(PNG)

S8 Fig Dynamics of biceps brachii perturbation. This figure shows the movement of the clavicle, as well as a bone of the finger and toe, in response to the perturbation of the biceps brachii. Data available at DOI:10.5281/zenodo.1069104.
(PNG)

S9 Fig Comparing models with and without bone weights and muscle strengths. The impact of the leg muscles was calculated with and without the addition of anatomical values for bone weight and muscle volume. These impacts were found to be significantly correlated with one another (F(1,25) = 6.83, R^2^ = 0.0214, *p* = 0.015), suggesting that at least in some portions of the body, our simplified network representation provides a reasonable approximation for more biophysically accurate network representations. Data available at DOI:10.5281/zenodo.1069104.
(PNG)

S10 Fig Probing musculoskeletal function for nonathletes. Recovery times were gathered for injuries to various muscles of nonathletes. We observed a significant correlation between muscle recovery time and impact deviation (F(1,14) = 5.02, R^2^ = 0.264, *p* = 0.041). Data available at DOI:10.5281/zenodo.1069104.
(PNG)

S11 Fig Correspondence of network topology and system function. Network topology, specifically average shortest path length, is significantly negatively correlated with the impact score estimated from the perturbative simulations of system dynamics (F(1,268) = 65.1, R^2^ = −0.4422, *p* < 0.0001). Data available at DOI:10.5281/zenodo.1069104.
(PNG)

S12 Fig Relation between musculoskeletal variation and muscular impact across two musculoskeletal networks. Here, we compare the percent change in impact score and degree for each muscle between the musculoskeletal network reported in the main text and that reported in the supplementary text. We observe that the impact score of muscles is more affected by larger changes in degree than by smaller changes in degree (F(1,268) = 5.76, R = 0.1450, *p* = 0.017). Data available at DOI:10.5281/zenodo.1069104.
(PNG)

S13 Fig Alternative perturbative approach. To establish a measure of impact per muscle hyperedge, objects were displaced into a fourth spatial dimension to avoid making arbitrary choices within three dimensions. An alternative approach would be to perturb each muscle in each of three orthogonal directions, calculating impact each time and calculating the vector sum of these three results. To answer the question of how these two approaches compare, we performed this experiment on the muscle-bone bipartite matrix to create two 270 × 1 vectors, one encoding the impact scores via displacement in the fourth dimension, and one encoding the vector sum of the three orthogonal displacements. The two vectors were significantly correlated with each other (F(1,268) = 1590, R^2^ = 0.856, *p* < 0.0001).
(PNG)
