# Supplementary material for: Structure, function, and control of the human musculoskeletal network
Source: PLoS Biol. 2018 Jan 18;16(1):e2002811. doi: 10.1371/journal.pbio.2002811 (PMC5773011; doi:10.1371/journal.pbio.2002811)
Supplement: S1 Text — This text file details construction of the alternative null models. (DOCX) [file pbio.2002811.s001.docx]

To probe the robustness of these results, two other null models were employed, the first of which was a random hypergraph approach. The random hypergraph is constructed first by randomly assigning edges, such that each muscle has degree of two, in order to account for the fact that each muscle in the real graph has degree of at least two. This results in a hypergraph composed of 540 connections. Because the true hypergraph has 1,012 connections, 472 additional edges are uniformly randomly assigned within the hypergraph (See S1 Table and S2 Table). The second null model was based on a random rewiring approach. In this method, each muscle is randomly rewired within the bipartite graph, such that each hyperedge maintains the same degree, but otherwise has connections assigned uniformly at random. This method also maintains the degree distribution of the overall bipartite graph (See S3 Table and S4 Table). S3 Fig shows a visual comparison between these models. Main results generated using these two alternate null models can be seen in S4 Fig. Finally, S5 Table shows results for a completely random null model, which does not preserve muscle degree but preserves total degree.
