# Supplementary material for: Structure, function, and control of the human musculoskeletal network
Source: PLoS Biol. 2018 Jan 18;16(1):e2002811. doi: 10.1371/journal.pbio.2002811 (PMC5773011; doi:10.1371/journal.pbio.2002811)
Supplement: S2 Text — This file provides a description of the choice of community detection resolution parameter. (DOCX) [file pbio.2002811.s002.docx]

To detect network communities, we performed a modularity maximization approach, as detailed in the Materials and methods section. Importantly, the modularity quality function includes a resolution parameter, γ, that can be used to tune the relative size of the communities: smaller values of γ identify larger communities and larger values of γ identify smaller communities (see S1 Fig and S2 Fig). We selected a single resolution parameter, γ = 4.3, for the analysis presented in the main text to generate 22 communities, equal to the number of categories in the motor homunculus. To show that our results are robust to reasonable variations in this choice, we generated communities with nearby resolution parameters γ = 4.2 and 4.4, which lead to the detection of 20 and 22 communities, respectively (S2 Fig). These two partitions were statistically similar to the original partition, as tested by the z-score of the Rand coefficient [47]: z = 105 for γ = 4.2 and z = 110 for γ = 4.4.
