# Supplementary material for: Structure, function, and control of the human musculoskeletal network
Source: PLoS Biol. 2018 Jan 18;16(1):e2002811. doi: 10.1371/journal.pbio.2002811 (PMC5773011; doi:10.1371/journal.pbio.2002811)
Supplement: S3 Text — This file provides a description of the alternative musculoskeletal network. (DOCX) [file pbio.2002811.s003.docx]

To ensure that our results are robust to differences across anatomical atlases, we constructed a second comprehensive musculoskeletal network from a different anatomical atlas [64] and assessed the reliability and reproducibility of our results. This exercise also addresses the notion that individual variation exists within musculoskeletal networks and enables us to ask whether our results hold over the two instances of normal variations captured within these two atlases. In addition to the alternative network being constructed from a different atlas, we also eliminated all non-bony nodes and adjusted the network, accordingly. For example, the interosseus membrane of the forearm was removed, and each connection to this membrane was replaced by two connections: one to the radius and one to the ulna. Our main results are reproduced in S6 Fig for this alternative network, and importantly, our findings remain consistent across both.
