# Supplementary material for: Structure, function, and control of the human musculoskeletal network
Source: PLoS Biol. 2018 Jan 18;16(1):e2002811. doi: 10.1371/journal.pbio.2002811 (PMC5773011; doi:10.1371/journal.pbio.2002811)
Supplement: S4 Text — (DOCX) [file pbio.2002811.s004.docx]

To provide insight into the dynamic model employed here, S8 Fig shows the trajectories of several bones over time, resulting from the perturbation of the biceps brachii. These example trajectories demonstrate that the clavicle, connected to many other muscles, is impacted much less than the bones of the hand. Bones of the hand are connected to the rest of the body through the elbow, which is flexed for the most part by the biceps brachii. The clavicle settles at a position about 50 units away from where it started, whereas the bones of the hand are moved nearly 250 units. Conversely, a bone very far away from the briceps brachii, in this case, a bone of the third toe, moves very little.
