# Supplementary material for: Structure, function, and control of the human musculoskeletal network
Source: PLoS Biol. 2018 Jan 18;16(1):e2002811. doi: 10.1371/journal.pbio.2002811 (PMC5773011; doi:10.1371/journal.pbio.2002811)
Supplement: S5 Text — (DOCX) [file pbio.2002811.s005.docx]

In the main text, bones were treated in the model as having equal weight and muscles were treated in the model as having equal strength. Intuitively, a more biophysically realistic network could be constructed that accounted for the physical properties of muscles and bones. While current data repositories, atlases, and anatomy texts do not provide complete information on all 173 bones and 270 muscles, we were able to combine information from multiple sources to collate a dataset of the weights of about 60% of the bones and the sizes of many of the muscles. Because the collation was quite time-consuming, we include this new dataset as Supporting information for others in the field to use for their own studies. We note that the sparsity of the data precluded a full-body simulation. However, we were able to find nearly complete data on the muscles and bones of the leg sub-network. This network included several of the lumbar vertebrae, hip bones, and distal bones up to the ankle (see bone weights in S7 Table, taken from [88,89]). In addition, we were able to locate data on the volume of many of the leg muscles from several sources [90–92], and we note that volume is thought to be a suitable estimator for muscle strength [93] (see muscle volumes in S6 Table). Muscle impact was calculated both with and without weights and volumes, and we found that the two estimates were significantly correlated with one another (S9 Fig). These results suggest that at least in some portions of the body, our simplified network representation provides a reasonable approximation for more biophysically accurate network representations.

References

88. Latimer HB, Lowrance E. Bilateral asymmetry in weight and in length of human bones. The Anatomical Record. 1965;152(2):217–224.

89. Gonc ̧alves D, d’Oliveira Coelho J, Acosta MA, Coelho C, Curate F, Ferreira MT, et al. One for all and all for one: Linear regression from the mass of individual bones to assess human skeletal mass completeness. American journal of physical anthropology. 2016;160(3):427–432.

90. Lube J, Cotofana S, Bechmann I, Milani TL, Özkurtul O, Sakai T, et al. Reference data on muscle volumes of healthy human pelvis and lower extremity muscles: an in vivo magnetic resonance imaging feasibility study. Surgical and Radiologic Anatomy. 2016;38(1):97–106.

91. Handsfield GG, Meyer CH, Hart JM, Abel MF, Blemker SS. Relationships of 35 lower limb muscles to height and body mass quantified using MRI. Journal of biomechanics. 2014;47(3):631–638.

92. Belavy D, Miokovic T, Rittweger J, Felsenberg D. Estimation of changes in volume of individual lower-limb muscles using magnetic resonance imaging (during bed-rest). Physiological measurement. 2010;32(1):35.

93. Akagi R, Takai Y, Ohta M, Kanehisa H, Kawakami Y, Fukunaga T. Muscle volume compared to cross-sectional area is more appropriate for evaluating muscle strength in young and elderly individuals. Age and ageing. 2009;38(5):564–569.
