# Supplementary material for: Structure, function, and control of the human musculoskeletal network
Source: PLoS Biol. 2018 Jan 18;16(1):e2002811. doi: 10.1371/journal.pbio.2002811 (PMC5773011; doi:10.1371/journal.pbio.2002811)
Supplement: S6 Text — (DOCX) [file pbio.2002811.s006.docx]

To demonstrate that the correlation between impact and recovery time generalizes to nonathletes, we gathered nonathletic injury recovery data. These data were culled from the large medical database, UpToDate. A review of this database revealed several recommended recovery times for nonathletes: 6 weeks for rotator cuff tears [94], 2 weeks for quadriceps injuries [95], 12 weeks for injuries to the calf muscles (gastrocnemius, soleus, and plantaris muscles) [96], and 4 weeks for adductor injuries (adductors brevis, longus, and magnus; gracilis; and obturator externus [97]). Using these data in nonathletes, we again observed a significant correlation between impact factor and recovery times (see S10 Fig). These supporting results suggest that the relation between impact factor and recovery time is conserved across athlete and nonathlete populations.

References

94. Management of rotator cuff tears. In: Post TW, editor. UpToDate. UpToDate; 2017.

95. Quadriceps muscle and tendon injuries. In: Post TW, editor. UpToDate. UpToDate; 2017.

96. Calf injuries not involving the Achilles tendon. In: Post TW, editor. UpToDate. UpToDate; 2017.

97. Adductor muscle and tendon injury. In: Post TW, editor. UpToDate. UpToDate; 2017.
