# Supplementary material for: Structure, function, and control of the human musculoskeletal network
Source: PLoS Biol. 2018 Jan 18;16(1):e2002811. doi: 10.1371/journal.pbio.2002811 (PMC5773011; doi:10.1371/journal.pbio.2002811)
Supplement: S7 Text — (DOCX) [file pbio.2002811.s007.docx]

It is important to understand to what degree the local network of muscles influences their impact deviation. Specifically, is there a local network structure that explains why one muscle has higher or lower impact than another muscle? To answer this question, we studied the local networks around each muscle from the muscle–muscle network in two different ways. First, we defined a minimal local network that included the target muscle and all muscles directly connected to it—that is, with paths of length 1. Second, we defined an expanded local network that included the target muscle and all muscles within two connections away—that is, with paths of length 2. We computed various summary statistics on each local muscle network, and we then assessed the degree to which those statistics were correlated with impact deviation. We chose network statistics that would quantify the degree of connectivity within the network in complementary ways. The first statistic that we studied was connection density, defined by the number of existing connections relative to the number of possible connections. The second statistic that we studied was the characteristic path length, defined by the average shortest path length in the network. Using the minimal local network, we found that both density and characteristic path length were weakly correlated with impact deviation (t = −1.78, DF = 127, Pearson’s R = −0.1274, *p* = 0.0382 and t = −2.33, DF = 127, R = −0.1569, *p* = 0.0105, respectively). Using the expanded local network, we found that both density and characteristic path length were strongly and significantly correlated with impact deviation (t = −7.4, DF = 124, R = −0.432, *p* < 0.0001 and t = −7.64, DF = 124, R = −0.4128, *p* < 0.0001, respectively). Notably, the correlations became even stronger as we expanded the local network further, to include paths of length 3 and 4. These data provide evidence that it is likely not the immediate local environment of the muscle that influences its impact most. Rather, it appears that factors influencing impact factor are spatially distributed. The nodes embedded in densely connected networks that are also able to reach other nodes in a few steps appear to be more impactful in the network.
