# Supplementary figures and images for: Structure, function, and control of the human musculoskeletal network
Source: PLoS Biol. 2018 Jan 18;16(1):e2002811. doi: 10.1371/journal.pbio.2002811 (PMC5773011; doi:10.1371/journal.pbio.2002811)

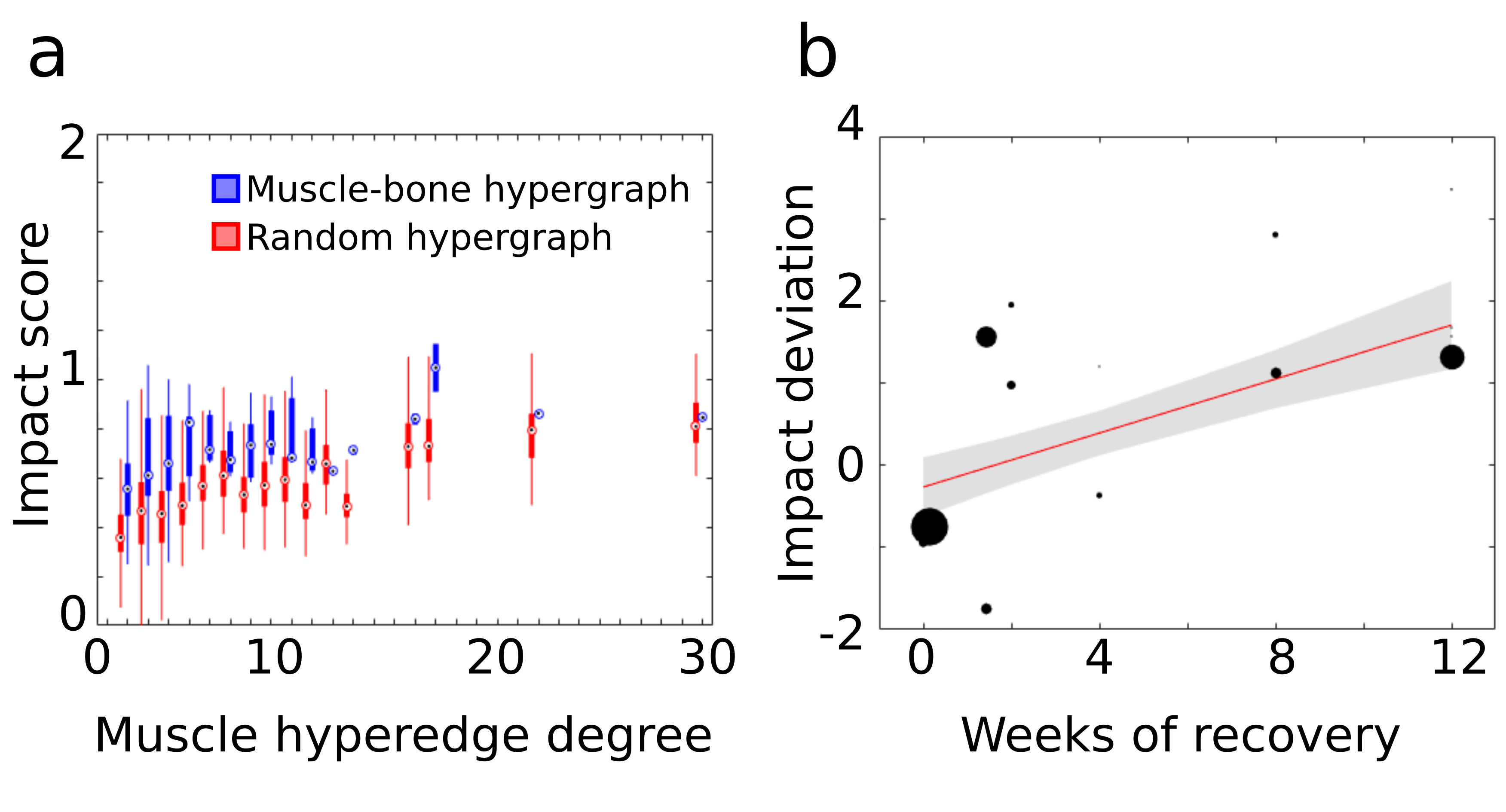

Supplement: S6 Fig — (a) The impact score plotted as a function of the hyperedge degree for a null hypergraph model and the observed musculoskeletal hypergraph. (b) Impact score deviation correlates with muscle recovery time following injury to muscles or muscle groups (F(1,12) = 40.2, p < 0.0001, R2 = 0.77). Shaded areas indicate 95% CIs, and data points are scaled according to the number of muscles included. Data available at DOI:10.5281/zenodo.1069104. (PNG) [file pbio.2002811.s023.png]

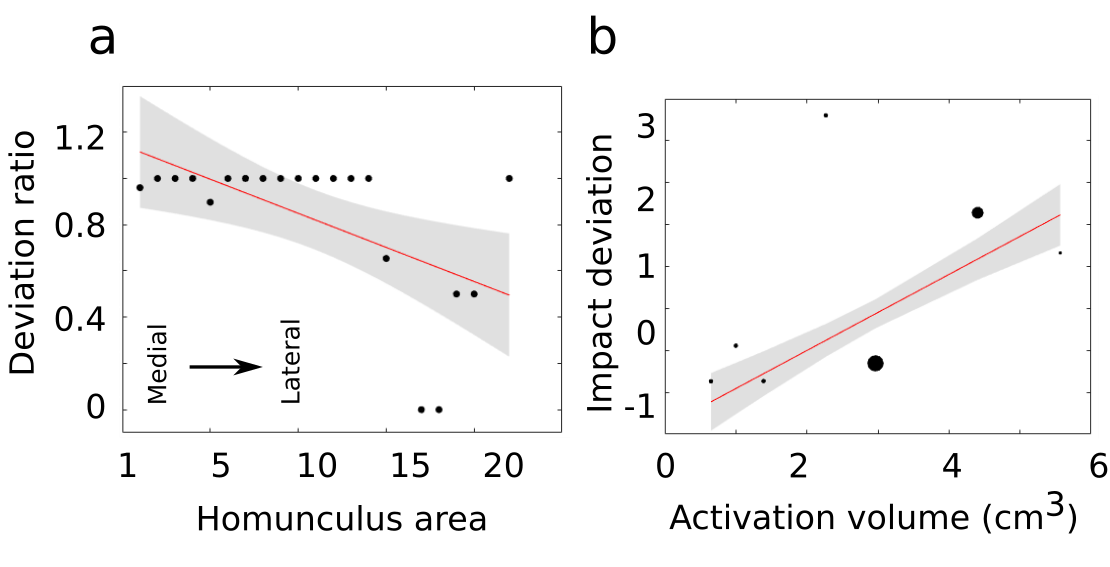

Supplement: S7 Fig — (a) Deviation ratio is significantly correlated with homuncular topology (F(1,18) = 8.88, R2 = 0.33, p = 0.0080), decreasing from medial (area 0) to lateral (area 22) regions. (b) Impact score deviation is significantly correlated with motor strip activation area (F(1,5) = 23.4, R2 = 0.82, p = 0.005). Data points are sized according to the number of muscles required for the particular movement. Data available at DOI:10.5281/zenodo.1069104. (PNG) [file pbio.2002811.s024.png]

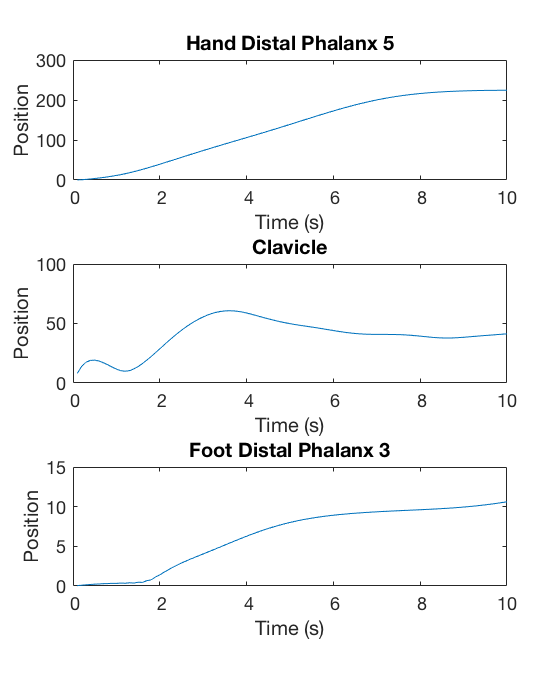

Supplement: S8 Fig — This figure shows the movement of the clavicle, as well as a bone of the finger and toe, in response to the perturbation of the biceps brachii. Data available at DOI:10.5281/zenodo.1069104. (PNG) [file pbio.2002811.s025.png]

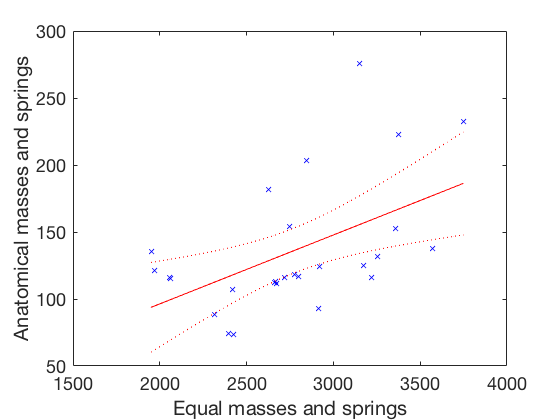

Supplement: S9 Fig — The impact of the leg muscles was calculated with and without the addition of anatomical values for bone weight and muscle volume. These impacts were found to be significantly correlated with one another (F(1,25) = 6.83, R2 = 0.0214, p = 0.015), suggesting that at least in some portions of the body, our simplified network representation provides a reasonable approximation for more biophysically accurate network representations. Data available at DOI:10.5281/zenodo.1069104. (PNG) [file pbio.2002811.s026.png]

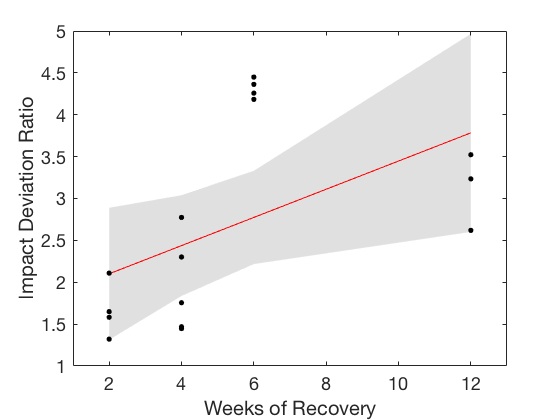

Supplement: S10 Fig — Recovery times were gathered for injuries to various muscles of nonathletes. We observed a significant correlation between muscle recovery time and impact deviation (F(1,14) = 5.02, R2 = 0.264, p = 0.041). Data available at DOI:10.5281/zenodo.1069104. (PNG) [file pbio.2002811.s027.png]

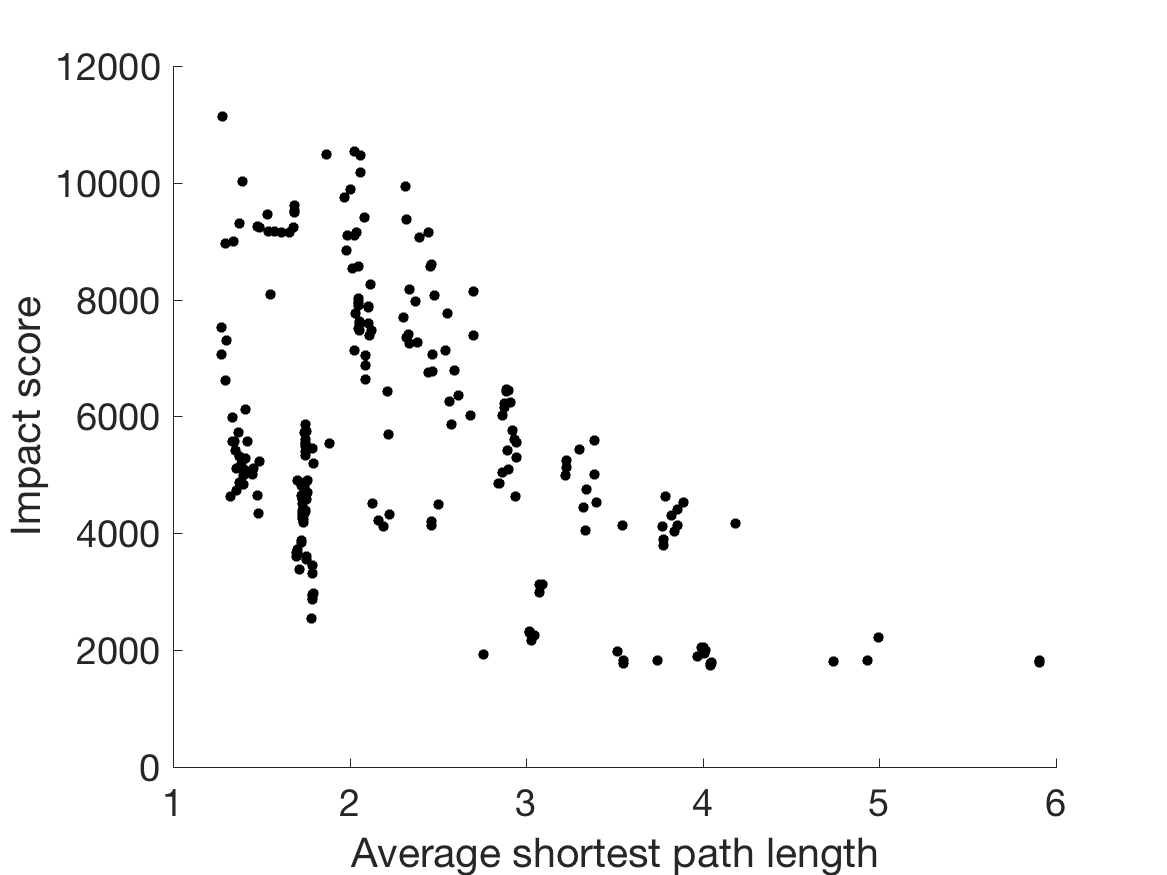

Supplement: S11 Fig — Network topology, specifically average shortest path length, is significantly negatively correlated with the impact score estimated from the perturbative simulations of system dynamics (F(1,268) = 65.1, R2 = −0.4422, p < 0.0001). Data available at DOI:10.5281/zenodo.1069104. (PNG) [file pbio.2002811.s028.png]

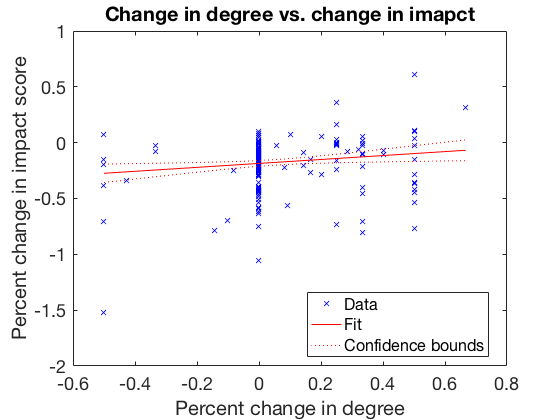

Supplement: S12 Fig — Here, we compare the percent change in impact score and degree for each muscle between the musculoskeletal network reported in the main text and that reported in the supplementary text. We observe that the impact score of muscles is more affected by larger changes in degree than by smaller changes in degree (F(1,268) = 5.76, R = 0.1450, p = 0.017). Data available at DOI:10.5281/zenodo.1069104. (PNG) [file pbio.2002811.s029.png]

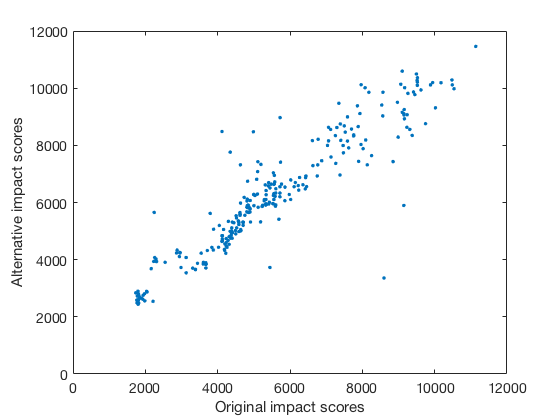

Supplement: S13 Fig — To establish a measure of impact per muscle hyperedge, objects were displaced into a fourth spatial dimension to avoid making arbitrary choices within three dimensions. An alternative approach would be to perturb each muscle in each of three orthogonal directions, calculating impact each time and calculating the vector sum of these three results. To answer the question of how these two approaches compare, we performed this experiment on the muscle-bone bipartite matrix to create two 270 × 1 vectors, one encoding the impact scores via displacement in the fourth dimension, and one encoding the vector sum of the three orthogonal displacements. The two vectors were significantly correlated with each other (F(1,268) = 1590, R2 = 0.856, p < 0.0001). (PNG) [file pbio.2002811.s030.png]
